# Supplementary material for: Myofibril and mitochondria morphogenesis are coordinated by a mechanical feedback mechanism in muscle
Source: Nat Commun. 2021 Apr 7;12:2091. doi: 10.1038/s41467-021-22058-7 (PMC8027795; doi:10.1038/s41467-021-22058-7)
Supplement: Supplementary file 1 — Supplementary Information [file 41467_2021_22058_MOESM1_ESM.pdf]

Supplementary Information

This file includes the Supplementary Figures from Avellaneda et al. 2021 including the legends, a Supplementary Table with a list of all fly strains and reagents, and the legends for the Supplementary Movies.

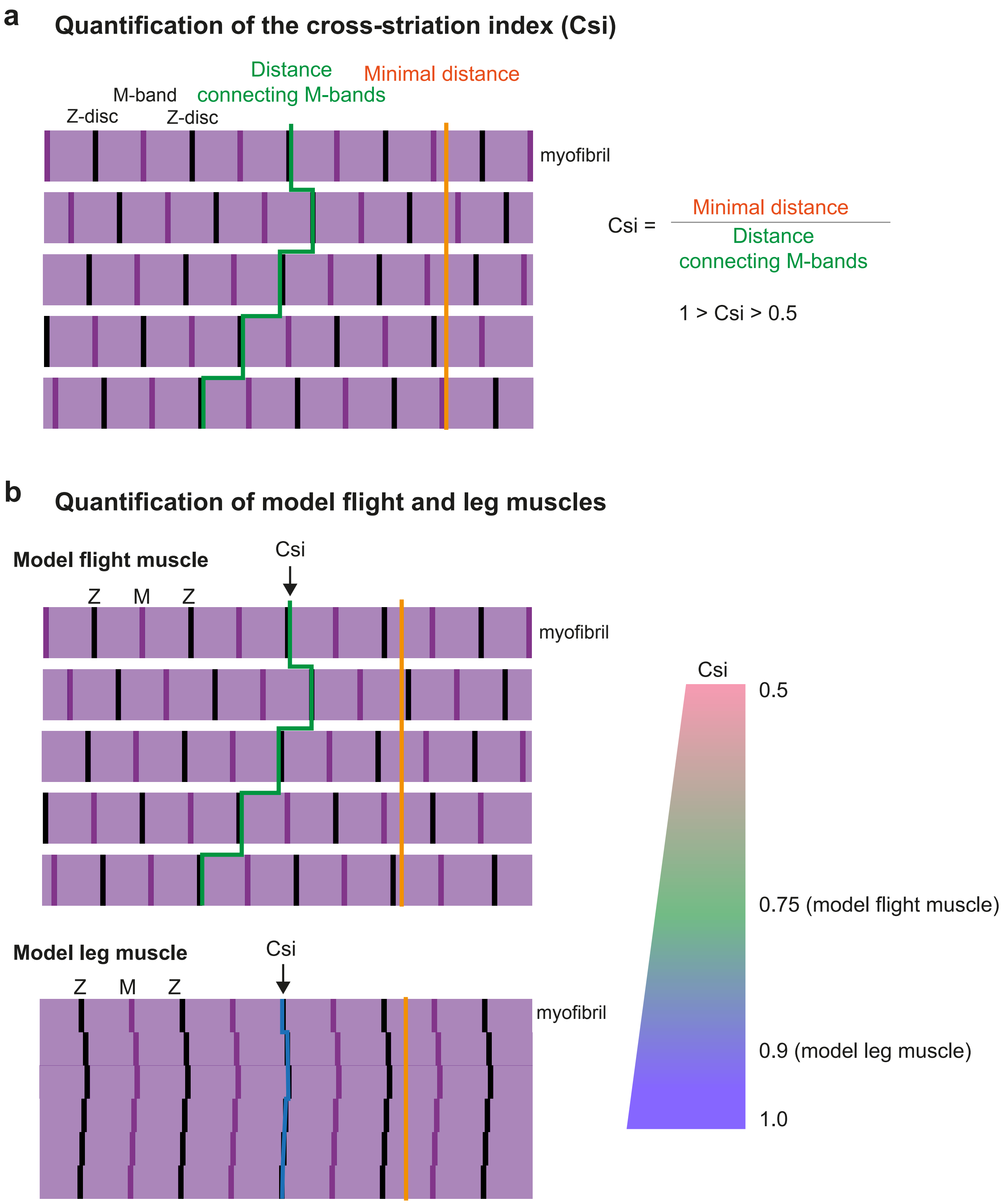

Supplementary Figure 1. Cross-striation index. (a) A cross-striation index (Csi) was defined as the ratio between the path length to connect M-bands (M) from adjacent myofibrils (green path) and the corresponding length of a straight line perpendicular to the myofibril axis (orange path). The closest M-band was chosen for the path connecting two myofibrils. Perfect alignment results in a Csi of 1, lower values represent progressively weaker alignment. **(b)** Representative examples for a Csi calculation in flight muscle (Csi about 0.75) and one in leg muscle (Csi about 0.9).

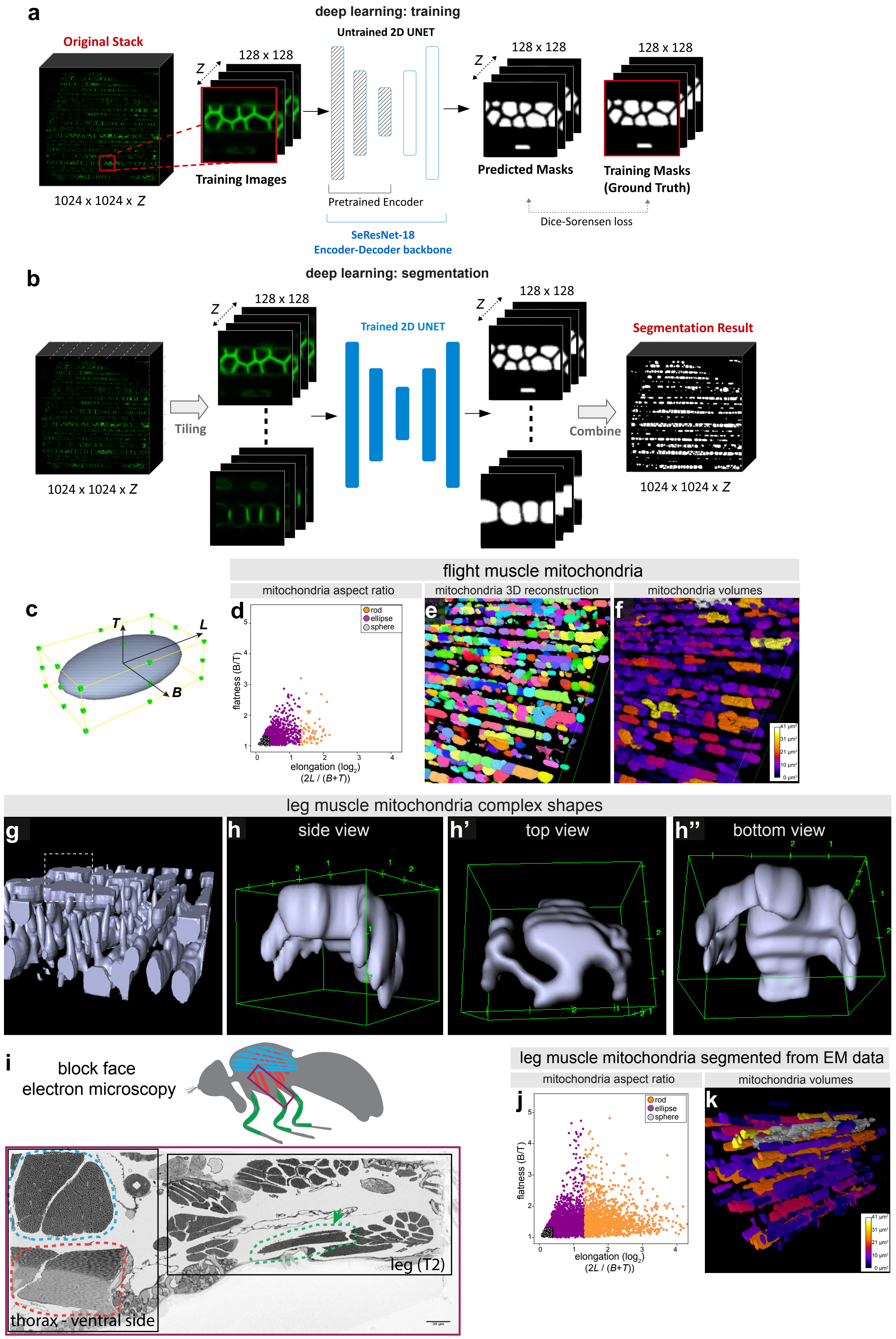

Supplementary Figure 2. Mitochondrial shape annotation. (a) Deep learning training: One small patch with 128x128 pixel resolution was extracted from one confocal stack (MOM-GFP labelled mitochondria) and was manually segmented using Fiji to generate a set of images and their corresponding ground truth masks.

Supplementary Figure 2 (cont.) Training has been achieved through a 2D UNET network architecture based on the SeResNet18 backbone with an encoder part pretrained on ImageNet Database using a Dice-Sorensen loss to optimize the network weights and Intersection Over Union (IoU) metrics to assess the segmentation quality.**(b)** Deep learning segmentation: four confocal stacks (MOM-GFP labelled mitochondria) with 1024x1024 pixel resolution were processed separately. For each of them, the whole stack was divided into 64 small 128x128 patches (tiling) and processed individually by the trained SeResNet18 2D UNET to generate a set of segmented patches which have further been combined to build the whole 1024x1024 stack segmentation result. **(c)** Diagram illustrating the long "*L*", middle "*B*" and short "*T*" axis of a model mitochondrion used for shape classification. The angle between the long axis relative to the myofibril axis was used to assign mitochondria orientation. **(d)** Morphology comparison on the basis of morphological descriptors, elongation and flatness of flight muscle; each dot represents an individual mitochondrion from a single 3D reconstruction (n = 1137 mitochondria, total muscle volume of 30,300 μm^3). **(e,f)** 3D segmentation of individual flight muscle mitochondria using Fiji, each mitochondrion was assigned a random colour (e, see Supplementary Movie 1), or was classified based on their volume (f). **(g,h)** 3D reconstruction of a cropped leg muscle volume shows complex mitochondria morphologies with thin extensions from surface and central mitochondria into the myofibril layer (g, Supplementary Movie 5). Side-, top- and bottom-views of an individual mitochondrion displaying its complex morphology (h-h'', Supplementary Movie 6). **(i)** diagram of DLM (blue) and DVM (red) flight and leg (green) muscles visible in a low magnification electron microscopy acquisition (magenta rectangle); the muscle used for the reconstruction shown in Fig. 2l-q, coxa muscle of the T2 leg, is highlighted by a green arrow. **(j)** Morphology comparison on the basis of morphological descriptors, elongation and flatness of leg muscle mitochondria; each dot represents an individual mitochondrion from a single 3D reconstruction (n = 1940 mitochondria, total muscle volume of 6308 μm^3). **(k)** 3D segmentation of individual leg muscle mitochondria from the SBF-EM acquisition where each mitochondrion was classified based on their volume.

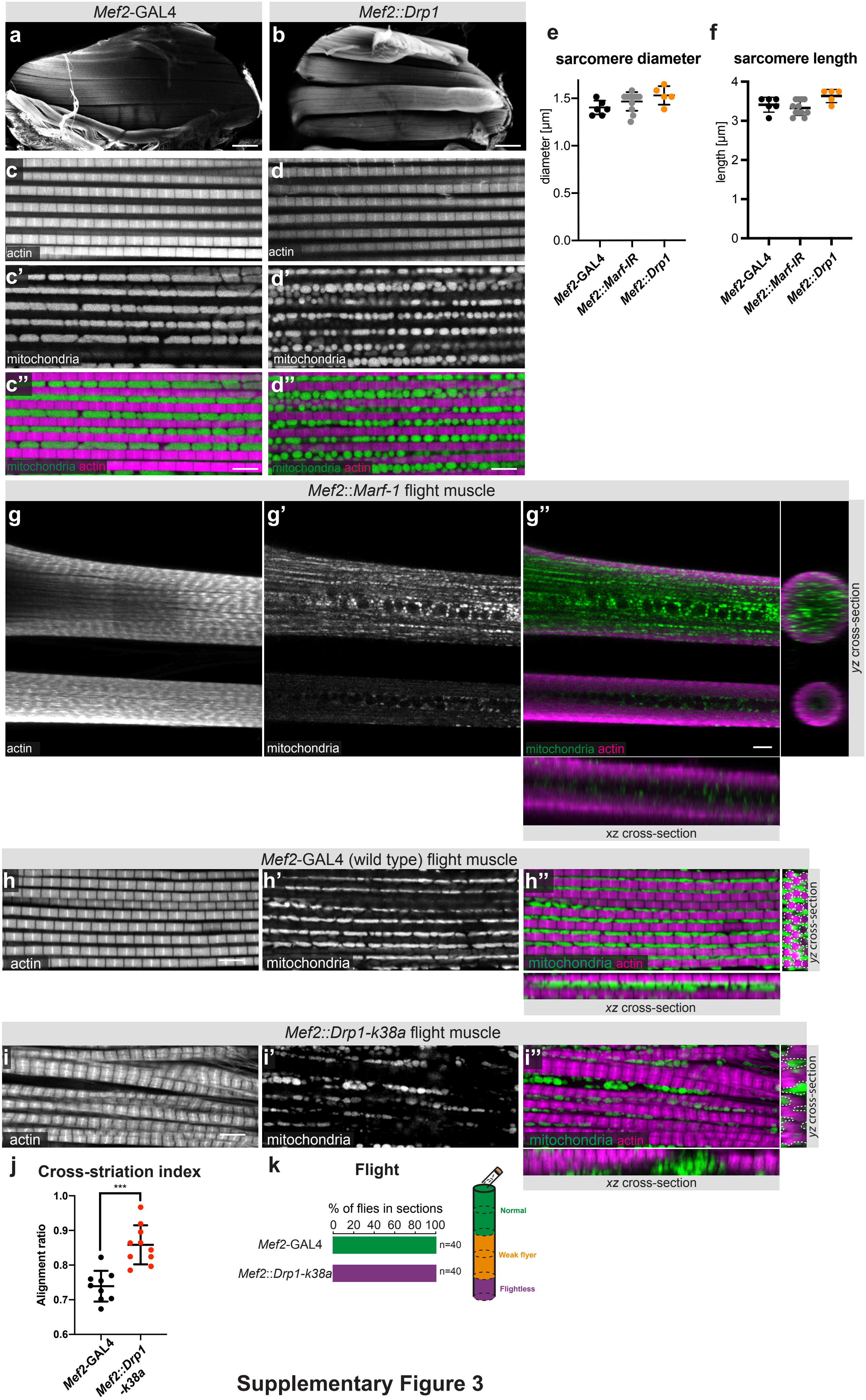

Supplementary Figure 3

Supplementary Figure 3. Manipulation of mitochondrial dynamics. (a-d) Adult hemi-thoraces (a,b) and flight muscles (c,d) from *Mef2*-GAL4 (a,c) and *Mef2::Drp1* (c,d), in which actin has been visualised with phalloidin and mitochondria with mito-GFP. Note the smaller round mitochondria upon *Drp1* over-expression (d). **(e,f)** Quantification of myofibril diameter (e; n = 6, 12, 5 animals, respectively) and sarcomere length (f; n = 6, 12, 5 animals, respectively) of the indicated genotypes. **(g)** Thick confocal stack displaying two *Mef2::Marf-1* flight muscles displaying a cross-striated tubular morphology. Note the centrally located mitochondria. **(h,i)** Adult flight muscles from *Mef2*-GAL4 (h) and *Mef2::Drp1-k38a* (i), in which actin has been visualised with phalloidin (h,i) and mitochondria with mito-GFP (h',i'). **(j)** Quantification of the lateral myofibril alignment using the cross-striation index in muscle of the indicated genotypes (j; n = 9, 10 animals, respectively; see Supplementary Fig. 1). Note the aligned myofibrils (i,j) and the flightless phenotype upon expression of *Drp1-k38a* **(k)**. In all plots the mean +/- standard-deviation (SD) is indicated, each dot the value from a single animal, and significance from two-tailed unpaired *t*-test is denoted as *p* = 0,0000920 (***). (n.s.) non-significant. Scale bars are 100 μm (a,b), 10 μm (g) or 5 μm (c,d,h,i).

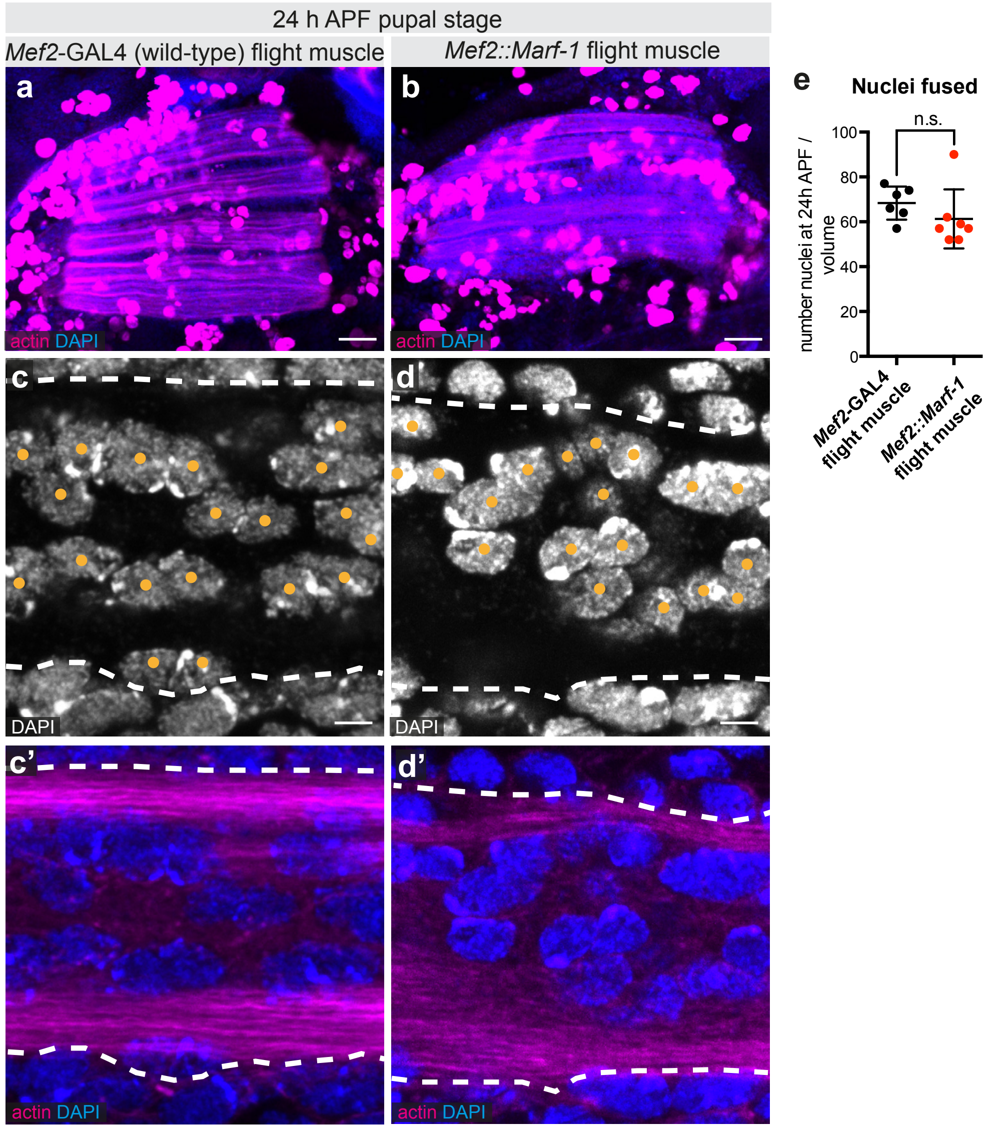

Supplementary Figure 4. Mitochondrial hyper-fusion does not block myoblast fusion. (a,b) Developing pupae at 24 h APF contain 6 DLM flight muscle fibers and show no obvious differences between *Mef2*-GAL4 wild type (a) and *Mef2::Marf-1* (b). **(c-e)** Individual nuclei were stained with DAPI and counted in an equal volume (11,000 µm^3) for all replicates from wild type (c) and *Mef2::Marf-1* (d). Phalloidin staining was used to mark the muscle fibers and distinguish nuclei of still unfused myoblasts from fused nuclei, marked by yellow dots. In (e) each dot represents the counts from one individual pupa (n=6 and 7 pupae, respectively), with the mean +/- standard-deviation (SD) indicated. Note that no significant difference (p-value = 0.2708 two-tailed unpaired t-test) in nuclei number is present. Scale bars are 40 µm (a,b) or 3 µm (c,d).

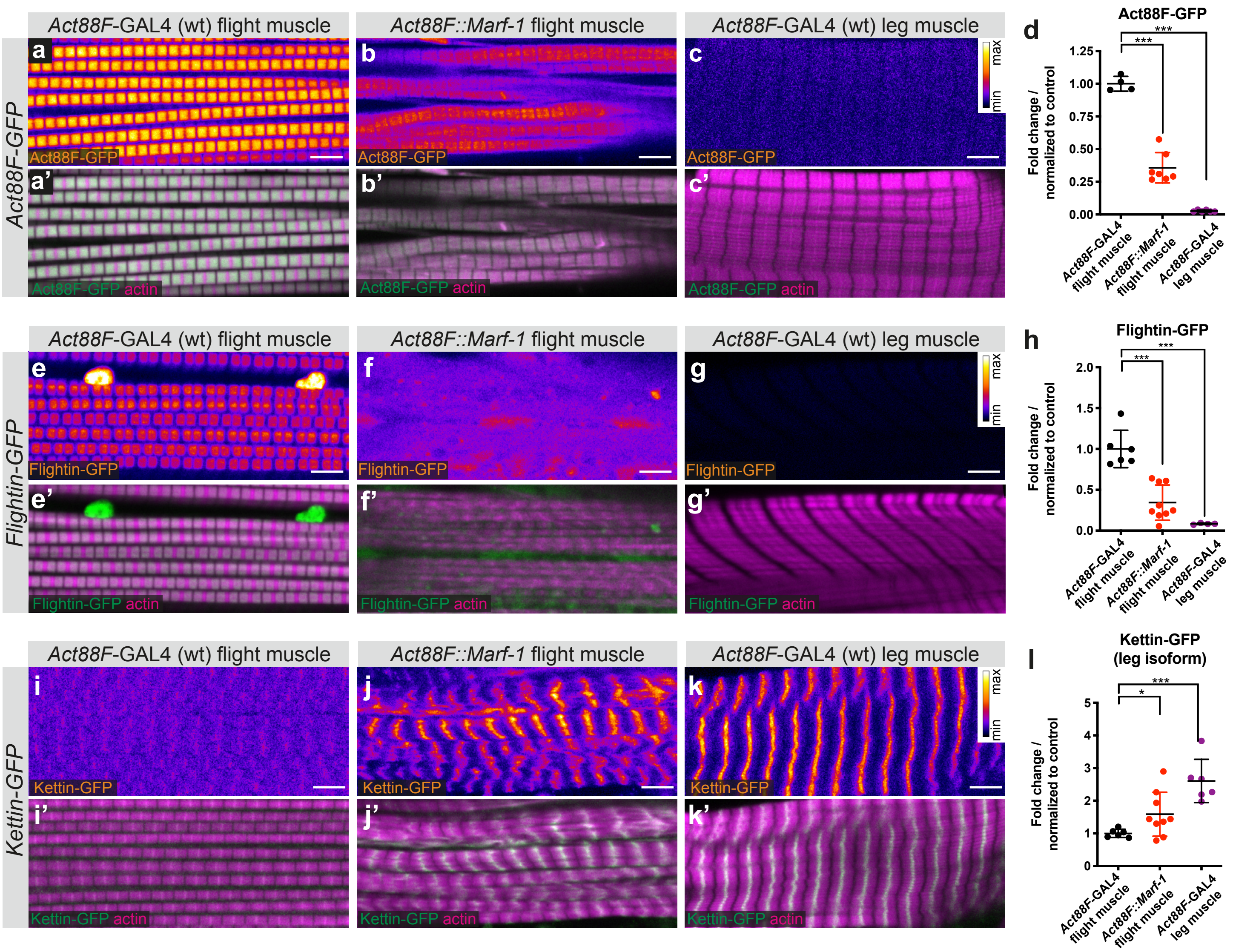

Supplementary Figure 5. Late stage mitochondria hyper-fusion causes a transcriptional shift to cross-striated muscle type. (a-k) Adult wild-type (a,e,i) as well as *Act88F::Marf-1* flight muscles (b,f,j) and wild-type leg muscles (c,g,k) expressing GFP-tagged muscle-type specific proteins Kettin-GFP (a-c), Actin 88F-GFP (e-g) and Flightin-GFP (i-k); samples were fixed and actin was visualised with phalloidin. Relative GFP fluorescence levels are represented via a pixel intensity scale (white represents higher intensity). **(d,h,l)** GFP fluorescence was quantified with quantitative confocal microscopy (see Methods) and plotted relative to control flight muscle levels (in (d) n = 6, 4 and 7 animals, respectively; in (h) n = 6, 4 and 9 animals, respectively; in (l) n = 6, 6 and 9 animals, respectively). Note that *Marf* over-expression in flight muscle converts the expression levels towards wild-type leg muscle levels. Mean +/- standard-deviation (SD) is indicated in all plots and significance from two-tailed unpaired Welch's *t*-tests is denoted as *p*-values ≤ 0,05 (*), *p* ≤ 0,001 (***). (n.s.) non-significant. Scale bars are 5 µm.

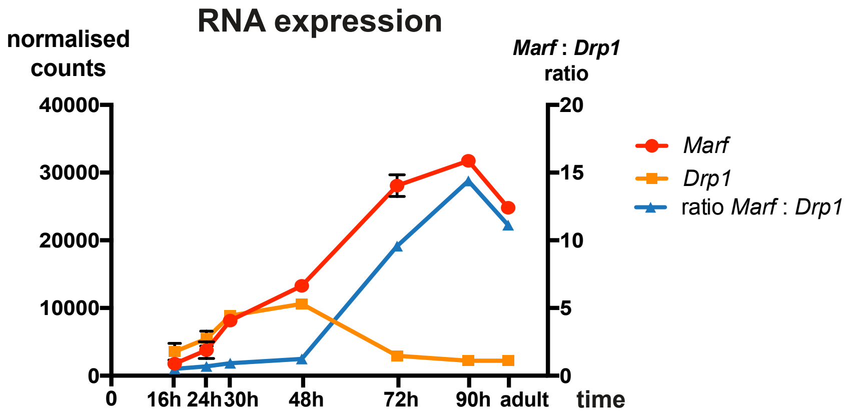

Supplementary Figure 6. Gene expression profiles of Marf and Drp1 during flight muscle development. *Marf* and *Drp1* transcript levels in flight muscles during pupal developmental time points until day 1 of adulthood. Data were extracted from a transcriptomics time course of isolated flight muscles; GEO number: GSE107247 (reference 36). Note that the ratio between *Marf:Drp1* stays equivalent until 32 h APF, whereas it shifts drastically to promote higher fusion after 48 h APF onwards. n = 2 for time points 30 h, 48 h, 90 h APF, adult flies, or n = 3 for time points 16 h, 24 h, 72 h APF; each "n" is an independent mRNA deep sequencing experiment, pooling mRNA from at least 150 pupae or flies each (reference 36); error bars denote +/-SD.

Supplementary Table 1. Key resources table - List of all fly strains and reagents used in this study.

Reagent type	Designation	Source	Identifiers	Additional information
Fly line	*UAS-Marf-1*	PMID: 29898954		Donor: Thomas Rival
Fly line	*UAS-Marf-2*	Bloomington	BDSC: 67157	Donor: Hugo J. Bellen
Fly line	*UAS-Drp1*	Bloomington	BDSC: 51647	Donor: Hugo J. Bellen
Fly line	*UAS-Drp1k38a*	PMID: 19056353	FBtp0041290	
Fly line	*Mef2*-GAL4	Bloomington	BDSC: 27390	
Fly line	*Act88F*-GAL4	PMID: 22008792		Source: Richard Cripps
Fly line	*him*-GAL4	PMID: 29846170		
Fly line	*1151*-GAL4	PMID: 9502718		Source: K VijayRaghavan
Fly line	*Act88F-GFP*	PMID: 26896675	VDRC: 318362	fTRG10028;
Fly line	*Kettin-GFP*	PMID: 26896675		fTRG569
Fly line	*Flightin-GFP*	PMID: 26896675	VDRC: 318238	fTRG876
Fly line	*UAS-Marf-IR*	PMID: 18799731		Source: Ming Guo
Fly line	*UAS-spalt-IR*	PMID: 22094701	VDRC: 13302	Flybase symbol: VDRC:v13302
Fly line	*UAS-mito-GFP*	Bloomington	BDSC: 25747	Donor Mark Peifer
Fly line	*UAS-MOM-GFP*	this paper		
Fly line	*UAS-Cherry-Gma*	PMID: 18184725		Source: Andrew Renault
Fly line	*w[1118]*	Bloomington	BDSC:3605	
Fly line	*Mhc[10]*	PMID: 2477306		Source: Sanford Bernstein
Antibody	mouse anti-complex-V(ATP5a)	abcam (15H4C4)	ab14748	(1: 500)
Antibody	rabbit anti-Spalt	PMID: 7905822		(1:100), Source Reinhard Schuh
Antibody	rat anti-Bruno	PMID:12591598		(1:1000) Source: Anne Ephrussi
Antibody	Alexa Fluor 488 goat anti-mouse	Invitrogen	A11001	(1: 500)
Antibody	Alexa Fluor 488 goat anti-rabbit	Invitrogen	A11034	(1: 500)
Chemical compound	Vectashield with DAPI	Vector laboratories	Ref H-1200	
Chemical compound	Vectashield	Vector laboratories	Ref H-1000	
Chemical compound	Schneider medium	Thermofisher	21720024	
Chemical compound	Rhodamine Phalloidin	Invitrogen (MolProbes)	R415	(1: 500)
Software, algorithm	Fiji (image J)	PMID: 22743772		
Software, algorithm	MyofibrilJ	PMID: 29846170		https://imagej.net/MyofibrilJ
Software, algorithm	MorpholibJ	PMID: 27412086		https://imagej.net/MorphoLibJ
Software, algorithm	Interactive Watershed (ImageJ)			https://imagej.net/Interactive_Watershed
Publicly available data source	mRNA-Seq data in Sup Fig 6	PMID: 29846170	GEO number: GSE107247	

		Sequence
Primer	sequence MOM-GFP	5’-[CGTGGTCAGCCATTAGAATG]-3’
Primer	sequence MOM-GFP	5’-[GCAGGCCGAATTCATGATTG]-3’
G-block	mitochondria outer membrane (MOM) targetting sequence	5'-ATGATTGAAATGAACAAAACTGCAATCGGCATTGCAGCGGGAGTAGCTGGAACTCTGTTTATTGGATACTGCATCTACTTCGACAAGAAGCGCCGCAGCGATCCCGAGTACAAGAAGAAAGTCCGT-3'

Supplementary Movie 1 (associated with Fig. 2). Animation from the rendering shown in Supplementary Fig. 2e of flight muscle mitochondria, distributed along the longitudinal axis of the myofibrils. Individual mitochondria are coloured randomly to highlight their spatial distribution.

Supplementary Movie 2 (associated with Fig. 2). Animation from the rendering in Fig. 2f-h showing the 3D reconstruction using images acquired each 40 nm via serial block-face electron microscopy. Individual mitochondria are shown with random colour and myofibrils appear during the movies in magenta. A single mitochondrion is highlighted in light pink together with myofibrils at the end of the movie.

Supplementary Movie 3 (associated with Fig. 2). Animation from the rendering shown in Fig. 2i. Leg muscle mitochondria are labelled with mito-GFP.

Supplementary Movie 4 (associated with Fig. 2). Animation from the rendering shown in Fig. 2k showing segmented leg muscle mitochondria. Separated mitochondria are coloured randomly.

Supplementary Movie 5 (associated with Fig. 2). Animation from the rendering shown in Supplementary Fig. 2g showing a close up of the leg muscle mitochondria network. Note the extensions that protrude from mitochondria into the space above and below where myofibrils are located (not represented).

Supplementary Movie 6 (associated with Fig. 2). Animation from the rendering shown in Supplementary Fig. 2h displaying the complex shape of a single leg muscle mitochondrion.

Supplementary Movie 7 (associated with Fig. 2). Animation from the rendering in Fig. 2m, p and q showing the 3D reconstruction of a leg muscle using images acquired each 30 nm via serial block-face electron microscopy. Individual mitochondria are shown with random colour. Individual mitochondria are highlighted at the end of the movie to illustrate their complexity and their thin channel protrusions towards the sarcomeric I-bands.

Supplementary Movie 8 (associated with Fig. 7). Montage of 4 animations from the 3D reconstructions shown in Fig. 7a,c,e,g displaying the mitochondria intercalation between assembled myofibrils from 24 h APF to 32 h APF. This phenomenon is blocked in *Mef2-::Marf-1* which leads to aggregated mitochondria that are unable to intercalate between myofibrils.

Supplementary Movie 9 (associated with Fig. 8): Montage of 4 animations from the 3D reconstructions shown in Fig. 8a,c,e,g displaying the mitochondria intercalated between myofibrils at 32h APF and 48h APF. Mitochondria in *Act88F::Marf-1* remain aggregated and are unable to intercalate between myofibrils.

Source Data file. Data from the analysis describing all number of animals and samples used for all the quantifications plotted in the figures. Where possible, individual values for the quantifications are presented, or averages per animal/sampled area, for each "n". Statistical analysis and *p*-value calculations are included.
