# Supplementary material for: Myofibril and mitochondria morphogenesis are coordinated by a mechanical feedback mechanism in muscle
Source: Nat Commun. 2021 Apr 7;12:2091. doi: 10.1038/s41467-021-22058-7 (PMC8027795; doi:10.1038/s41467-021-22058-7)
Supplement: Supplementary file 3 — Description of Additional Supplementary Files [file 41467_2021_22058_MOESM3_ESM.pdf]

Description of Additional Supplementary Files

File Name: Supplementary Movie 1
Description: **(associated with Fig. 2).** Animation from the rendering shown in Supplementary Fig. 2e of flight muscle mitochondria, distributed along the longitudinal axis of the myofibrils. Individual mitochondria are coloured randomly to highlight their spatial distribution.

File Name: Supplementary Movie 2
Description: **(associated with Fig. 2).** Animation from the rendering in Fig. 2f-h showing the 3D reconstruction using images acquired each 40 nm via serial block-face electron microscopy. Individual mitochondria are shown with random colour and myofibrils appear during the movies in magenta. A single mitochondrion is highlighted in light pink together with myofibrils at the end of the movie.

File Name: Supplementary Movie 3
Description: **(associated with Fig. 2).** Animation from the rendering shown in Fig. 2i. Leg muscle mitochondria are labelled with mito-GFP.

File Name: Supplementary Movie 4
Description: **(associated with Fig. 2).** Animation from the rendering shown in Fig. 2k showing segmented leg muscle mitochondria. Separated mitochondria are coloured randomly.

File Name: Supplementary Movie 5
Description: **(associated with Fig. 2).** Animation from the rendering shown in Supplementary Fig. 2g showing a close up of the leg muscle mitochondria network. Note the extensions that protrude from mitochondria into the space above and below where myofibrils are located (not represented).

File Name: Supplementary Movie 6
Description: **(associated with Fig. 2).** Animation from the rendering shown in Supplementary Fig. 2h displaying the complex shape of a single leg muscle mitochondrion.

File Name: Supplementary Movie 7
Description**: (associated with Fig. 2).** Animation from the rendering in Fig. 2m, p and q showing the 3D reconstruction of a leg muscle using images acquired each 30 nm via serial block-face electron microscopy. Individual mitochondria are shown with random colour. Individual mitochondria are highlighted at the end of the movie to illustrate their complexity and their thin channel protrusions towards the sarcomeric I-bands.

File Name: Supplementary Movie 8
Description: **(associated with Fig. 7).** Montage of 4 animations from the 3D reconstructions shown in Fig. 7a,c,e,g displaying the mitochondria intercalation between assembled myofibrils from 24 h APF to 32 h APF. This phenomenon is blocked in Mef2- ::Marf-1 which leads to aggregated mitochondria that are unable to intercalate between myofibrils.

File Name: Supplementary Movie 9
Description: **(associated with Fig. 8):** Montage of 4 animations from the 3D reconstructions shown in Fig. 8a,c,e,g displaying the mitochondria intercalated between myofibrils at 32h APF and 48h APF.

Mitochondria in Act88F::Marf-1 remain aggregated and are unable to intercalate between myofibrils.
