# Supplementary material for: Myofibril and mitochondria morphogenesis are coordinated by a mechanical feedback mechanism in muscle
Source: Nat Commun. 2021 Apr 7;12:2091. doi: 10.1038/s41467-021-22058-7 (PMC8027795; doi:10.1038/s41467-021-22058-7)
Supplement: Supplementary file 13 — Reporting Summary [file 41467_2021_22058_MOESM13_ESM.pdf]

nature research

Corresponding author(s): Nuno M Luis, Frank Schnorrer

Last updated by author(s): Jan 31, 2021

Reporting Summary

Nature Research wishes to improve the reproducibility of the work that we publish. This form provides structure for consistency and transparency in reporting. For further information on Nature Research policies, see our Editorial Policies and the Editorial Policy Checklist.

Statistics

For all statistical analyses, confirm that the following items are present in the figure legend, table legend, main text, or Methods section.

n/a	Confirmed	
☐	☒	The exact sample size (n) for each experimental group/condition, given as a discrete number and unit of measurement
☐	☒	A statement on whether measurements were taken from distinct samples or whether the same sample was measured repeatedly
☐	☒	The statistical test(s) used AND whether they are one- or two-sided<br>*Only common tests should be described solely by name; describe more complex techniques in the Methods section.*
☒	☐	A description of all covariates tested
☒	☐	A description of any assumptions or corrections, such as tests of normality and adjustment for multiple comparisons
☐	☒	A full description of the statistical parameters including central tendency (e.g. means) or other basic estimates (e.g. regression coefficient) AND variation (e.g. standard deviation) or associated estimates of uncertainty (e.g. confidence intervals)
☐	☒	For null hypothesis testing, the test statistic (e.g. F, t, r) with confidence intervals, effect sizes, degrees of freedom and P value noted<br>*Give P values as exact values whenever suitable.*
☒	☐	For Bayesian analysis, information on the choice of priors and Markov chain Monte Carlo settings
☒	☐	For hierarchical and complex designs, identification of the appropriate level for tests and full reporting of outcomes
☒	☐	Estimates of effect sizes (e.g. Cohen's d, Pearson's r), indicating how they were calculated

Our web collection on statistics for biologists contains articles on many of the points above.

Software and code

Policy information about availability of computer code

Data collection	No software was used for data collection.
Data analysis	A Github Repository containing the code used for the Deep Learning Segmentation and the Shape classification and quantification analysis, including a detailed Readme file and notes directly into the notebooks, can be found at https://github.com/fabda/Myofibril_paper. Fiji (ImageJ 2.1.0/1.53c), a freely available, open source software, published plugins: MyofibrilJ, MorpholibJ, Interactive Watershed; segmentation of the serial block-face EM data was done with open source software IMOD v. 4.7.12 (https://bio3d.colorado.edu/imod/).

For manuscripts utilizing custom algorithms or software that are central to the research but not yet described in published literature, software must be made available to editors and reviewers. We strongly encourage code deposition in a community repository (e.g. GitHub). See the Nature Research guidelines for submitting code & software for further information.

Data

Policy information about availability of data

All manuscripts must include a data availability statement. This statement should provide the following information, where applicable:

- Accession codes, unique identifiers, or web links for publicly available datasets
- A list of figures that have associated raw data
- A description of any restrictions on data availability

UNET architectures were used with ImageNet pre-trained seResNet18 and ResNet152 encoders as backbones to generate Deep Learning model for mitochondria segmentation. The gene expression data of indirect flight muscle development was published previously and is available at NCBI's Gene Expression Omnibus (GEO) with the accession number GSE107247. Confocal raw datasets generated during the current study are available from the corresponding authors upon reasonable request.

Field-specific reporting

Please select the one below that is the best fit for your research. If you are not sure, read the appropriate sections before making your selection.

- [x] Life sciences
- [ ] Behavioural & social sciences
- [ ] Ecological, evolutionary & environmental sciences

For a reference copy of the document with all sections, see nature.com/documents/nr-reporting-summary-flat.pdf

Life sciences study design

All studies must disclose on these points even when the disclosure is negative.

Sample size	Sample sizes differed from experiment to experiment, depending on the number of animals generated. All data points collected from the available animals were used to generate data and all are included in the analysis, as detailed in the Source Data file.
Data exclusions	No data was excluded from the analysis.
Replication	For all experiments at least 2 independent experiments were conducted, which showed reproducibility of the phenotype as reported. Each sample quantified relates to an individual animal, thus every sample is a biological replicate, and panels show an aggregate of all independent experiments quantified.
Randomization	Each group compared was the progeny generated by the crossing of independent progenitors, thus no randomization was needed.
Blinding	Investigators were not blind to provenance of the data analyzed - the investigator setting up the experiment was in most cases the same collecting the samples and performing the analysis, thus blinding was not possible. In almost all panels the entire dataset was used for the analysis and the same procedure applied to all samples. An exception are the "cross-striation index" were lines are drawn manually by the investigator; we increased the number of areas to quantify as much as possible to negate the effects of selection bias.

Reporting for specific materials, systems and methods

We require information from authors about some types of materials, experimental systems and methods used in many studies. Here, indicate whether each material, system or method listed is relevant to your study. If you are not sure if a list item applies to your research, read the appropriate section before selecting a response.

Materials & experimental systems

n/a	Involved in the study	
[ ]	[x]	Antibodies
[x]	[ ]	Eukaryotic cell lines
[x]	[ ]	Palaeontology and archaeology
[ ]	[x]	Animals and other organisms
[x]	[ ]	Human research participants
[x]	[ ]	Clinical data
[x]	[ ]	Dual use research of concern

Methods

n/a	Involved in the study	
[x]	[ ]	ChIP-seq
[x]	[ ]	Flow cytometry
[x]	[ ]	MRI-based neuroimaging

Antibodies

Antibodies used	mouse anti-complex-V(ATP5a) abcam ab14748 clone: 15H4C4 lot: GR114324-19 ; rabbit anti-Spalt PMID: 7905822; Alexa Fluor 488 goat anti-mouse Invitrogen A11001 lot:1834337; Alexa Fluor 488 goat anti-rabbit Invitrogen A11034 lot: 1705912; rat anti-Bruno PMID:12591598
Validation	anti-ATP5a (ab14748) validated in PMID: 27529784 and PMID: 25428350. anti-Spalt validated in PMID: 7905822 and PMID: 22094701. anti-Bruno validated in PMID:12591598 and PMID: 25532219;

Animals and other organisms

Policy information about studies involving animals; ARRIVE guidelines recommended for reporting animal research

Laboratory animals	Drosophila melanogaster strains as indicated in the Methods section. Adult flies dissected for microscopy observation were 2-3 days old. The flight tests were performed on 7 days old adult animals. All adult flies use in this paper are males, excluding progeny from Act88F-G4 and 1151-G4 progenitors as the transgene is inserted in the X chromosome. For developmental experiments in pupal stages animals from both sexes were used indistinguishably.
Wild animals	The study did not involve wild animals.

Field-collected samples	The study did not involve samples collected from the field.
Ethics oversight	No ethical approval necessary as no vertebrate studies involved

Note that full information on the approval of the study protocol must also be provided in the manuscript.
